# Assessing Quality of Life in Spinal Surgery

**DOI:** 10.5812/aapm.3552

**Published:** 2012-04-01

**Authors:** Bartosz Godlewski

**Affiliations:** 1 Department of Neurosurgery and Peripheral Nerves Surgery, Medical University of Lodz, Lodz, Poland

**Keywords:** Surgery, Quality of Life, Outcome Assessment (Health Care)

Dear Editor,

I have read with interest the article by Farzanegan et al. (1). The authors focused on a very important aspect of quality-of-life assessment in lumbar discopathy patients following neurosurgical procedures. In their daily clinical practice, neurosurgeons by and large concentrate only on the presence of neurological deficits (paresis or paralysis) and pain, while ignoring the social aspect of recovery. This is partly due to the nature of work of the operating surgeon and associated time constraints, and partly to the prevailing patterns of clinical care and the belief that further treatment should rest with other medical professionals, mostly rehabilitation specialists. The use of quality-of-life scales that account for the patient’s psychological well-being and social aspects appears to have a significant impact on comprehensive evaluation of treatment outcomes. Assessment of neurological deficits and muscle strength is mostly based on the Frankel neurological performance scale and the ASIA impairment scale (2, 3). Evaluation of pain intensity commonly relies on visual analog scales (VAS) (4). Quality of life is popularly assessed with the Oswestry Disability Index, which serves to determine the degree of disability related to thoracic and lumbosacral spine problems (5, 6), while the neck disability index (NDI) form is used in the case of patients with cervical spine pathology (7, 8).

The authors used the 36-item short-form health survey (SF-36) (9), which assesses health-related quality of life, to evaluate their patients before surgery and at 6 and 12 months post-surgery. They also accounted for a number of other factors directly influencing health, such as social and demographic factors. Collating such data required considerable precision and systematic work. The results reveal statistically significant improvements in health status after the surgery compared to pre-operative data (1).

The final outcome of surgery is influenced by a number of factors. In my opinion, the most important determinants of treatment success are the choice of an appropriate surgical technique according to the specific needs of the patient and performing a technically correct procedure without complications. However, mental and social aspects of recovery should not be neglected. Each patient needs a sincere talk, an explanation of the details of the treatment plan, a discussion of the expected benefits and surgery-related risks. The patient must also be certain that he or she will not be left alone should problems arise in the post-operative period and that help will be provided. This approach helps achieve better long-term treatment outcomes reflected in quality-of-life scales.

In conclusion, the optimum approach to a comprehensive evaluation of the health of patients following spinal surgery would be to simultaneously apply traditional scoring systems for neurological status and pain and methods assessing the quality of life.
